# Discharge instructions given to women following delivery by cesarean section in Sub-Saharan Africa: A scoping review

**DOI:** 10.1371/journal.pgph.0000318

**Published:** 2022-04-22

**Authors:** Juliet Musabeyezu, Jenna Santos, Anne Niyigena, Ange Uwimana, Bethany Hedt-Gauthier, Adeline A. Boatin

**Affiliations:** 1 Harvard Medical School, Boston, MA, United States of America; 2 Boston College, Boston, MA, United States of America; 3 Partners in Health, Kigali, Rwanda; 4 University of Illinois College of Medicine, Chicago, IL, United States of America; 5 Department of Global Health and Social Medicine, Harvard Medical School, Boston, MA, United States of America; 6 Program in Global Surgery and Social Change, Harvard Medical School, Boston, MA, United States of America; 7 Department of Obstetrics & Gynecology, Massachusetts General Hospital, Boston, MA, United States of America; 8 Center for Global Health, Massachusetts General Hospital, Boston, MA, United States of America; Centre for Sexual Health & HIV/AIDS Research (CeSHHAR)R, ZIMBABWE

## Abstract

**Objective:**

A scoping review of discharge instructions for women undergoing cesarean section (c-section) in sub-Saharan Africa (SSA).

**Method:**

Studies were identified from PubMed, Globus Index Medicus, NiPAD, EMBASE, and EBSCO databases. Eligible papers included research based in a SSA country, published in English or French, and containing information on discharge instructions addressing general postnatal care, wound care, planning of future births, or postpartum depression targeted for women delivering by c-section. For analysis, we used the PRISMA guidelines for scoping reviews followed by a narrative synthesis. We assessed quality of evidence using the GRADE system.

**Results:**

We identified 78 eligible studies; 5 papers directly studied discharge protocols and 73 included information on discharge instructions in the context of a different study objective. 37 studies addressed wound care, with recommendations to return to a health facility for dressing changes and wound checks between 3 days to 6 weeks. 16 studies recommended antibiotic use at discharge, with 5 specifying a particular antibiotic. 19 studies provided recommendations around contraception and family planning, with 6 highlighting intrauterine device placement immediately after birth or 6-weeks postpartum and 6 studies discussing the importance of counselling services. Only 5 studies provided recommendations for the evaluation and management of postpartum depression in c-section patients; these studies screened for depression at 4–8 weeks postpartum and highlighted connections between c-section delivery and the loss of self-esteem as well as connections between emergency c-section delivery and psychiatric morbidity.

**Conclusion:**

Few studies in SSA directly examine discharge protocols and instructions for women following c-section. Those available demonstrate wide variation in recommendations. Research is needed to develop structured evidence-based instructions with clear timelines for women. These instructions should account for financial burden, access to resources, and education of patients and communities.

## Introduction

Cesarean section (c-section) is the most common surgery in the world [1], and in sub-Saharan Africa around 7.3% of births occur by c-section [2]. Complications such as infection remain higher for those who have undergone c-section than for those who have delivered by vaginal birth [3]. This is most apparent in sub-Saharan Africa (SSA}, where maternal mortality following c-section is 50 times greater than high income countries [4]. Although most studies examining postpartum complications are limited to outcomes up to the point of discharge, complications can also occur in the period after, with studies showing that superficial surgical site infections after c-section are most likely to manifest after a woman has been discharged [5–7].

Moreover, even in the context of normal healing and recovery, compared to women delivering vaginally, women delivering by c-section also face different challenges for wound healing, among these planning around future births and functional recovery are critical. Women who have had one c-section will be at an increased risk of needing another c-section during their next pregnancy, with gestational age, maternal obesity, and underlying medical conditions being key elements in decision-making around whether a woman is offered trial of labor versus a repeat c-section [8, 9]. Therefore, counselling around contraceptive use and access to family planning in the recovery period after c-section can play an instrumental role in the management of future pregnancies. Separately, assessing and managing postpartum depression in the weeks following delivery is a pivotal element of comprehensive postpartum care. Studies from SSA indicate rates of major depressive disorder in the postpartum period as high as 10% [10] with some studies suggesting higher rates among women delivering by c-section [11–13].

Collectively, the period following delivery is a prime opportunity to address some of these challenges, starting with the instructions that women receive when they leave the hospital. While the World Health Organization (WHO) provides protocols for antenatal and perinatal care [14–16], there are no standardized protocols for the postnatal period targeting women who deliver by c-section. This scoping review aims to contribute to the development of such protocols. Specifically, this project will address the question: “What discharge instructions are given to c-section patients in sub-Saharan Africa regarding wound care, planning of future births, and postpartum depression?”.

## Methods

Our team conducted a scoping review following the five stages outlined in the Arksey and O’Malley framework [17]. This review was undertaken by a team of 6 researchers, possessing collective expertise in research methods, data analysis, surgical care, and obstetrics and gynecology.

### Stage 1: Identifying research questions

A PICOCS framework was used to develop a clear research question. Specifically, we aimed to answer the following questions: “what discharge instructions are given to women delivered by c-section in sub-Saharan Africa in 3 domain areas: 1) wound care 2) planning of future births and contraception, and 3) postpartum depression”

### Stage 2: Identification of literature sources

For the purposes of this study, we defined “c-section” as the surgical delivery of a baby through an incision in the abdomen and uterus [18], and “sub-Saharan Africa” as all the African countries located partially or completely south of the Sahara, including Sudan and Mauritania [19]. Based on these definitions, we compiled a list of related search terms that encompassed the definition and associated synonyms of both of these primary concepts. A full list of search terms is available in Table A in S1 Text. We searched PubMed, Global Index Medicus, NiPaD, EMBASE and Global Health for all studies that included the glossary of terms relevant to “c-section” and “sub-Saharan Africa” in their title and abstract from inception of databases until August 5^th^, 2020. We also used this glossary of search terms to scan the websites of all 47 sub-Saharan health ministries and the websites of professional obstetrical societies such as ACOG, WHO, SASOG, AFOG, RCOG, FIGO, WACS, ECSACOG for documents or web pages with information pertinent to “c-section” and “sub-Saharan Africa”.

### Stage 3: Selection of literature sources

We first screened articles at the title and abstract level using Covidence, an online data base service that provides a platform for systematic reviews, with independent reviews by at least two researchers (JM, JS, AN). At this level, we included studies that reported on either c-section patients as the population of focus or both c-section and vaginal birth patients. Included studies had sub-Saharan Africa as the geographical setting. Only studies in English or French were included. We excluded studies that were uniquely focused on inpatient perinatal maternal mortality or neonatal outcomes following cesarean section, as they were considered irrelevant to the post-discharge period. The full text manuscripts for studies meeting eligibility were retrieved. Where articles could not be procured through library resources, authors were emailed for access. Where this was not possible, articles were excluded.

**Fig 1:**
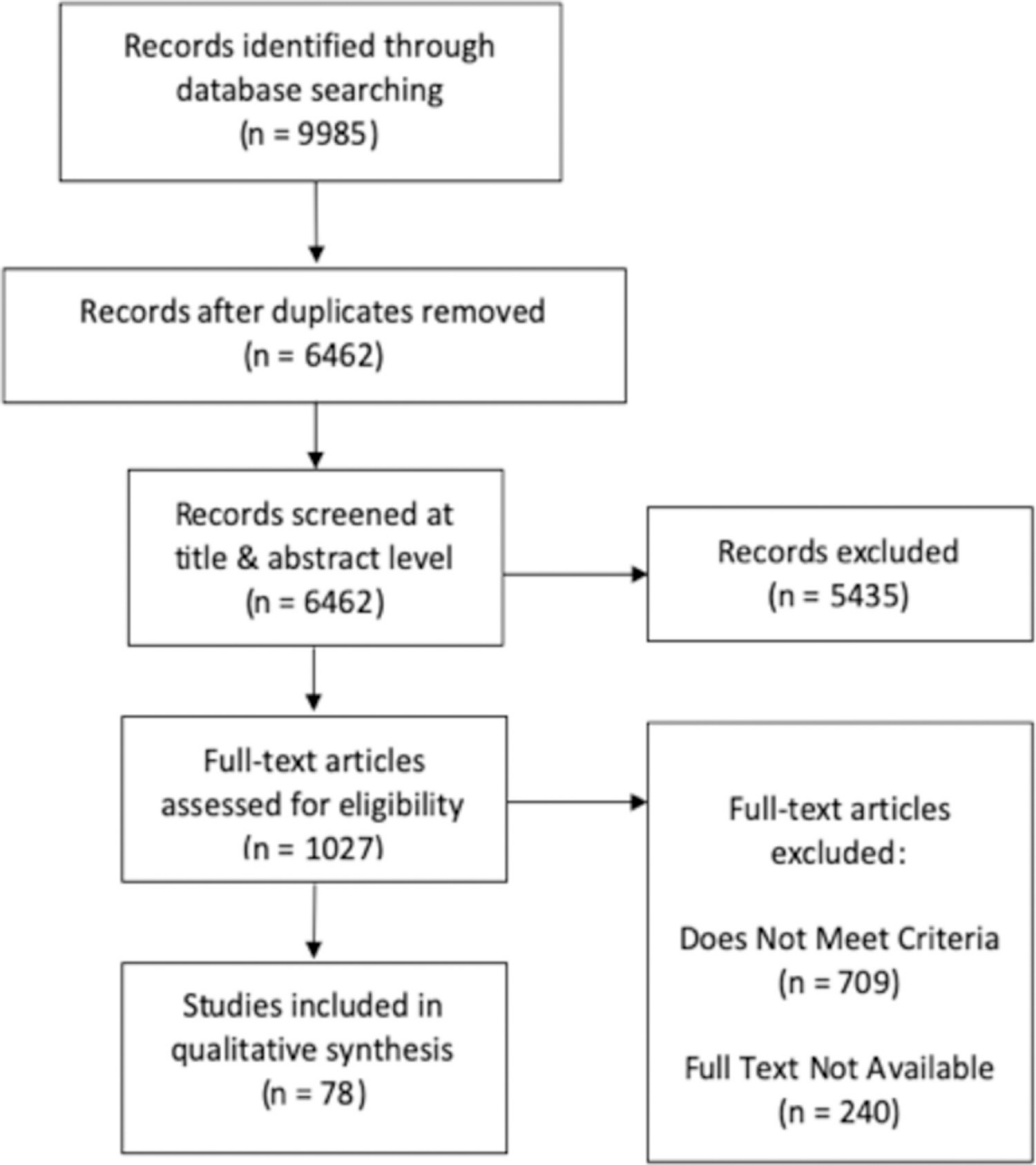
Study selection process.

Retrieved full text articles were further screened against our study objective. Specifically, articles were included if they provided discharge instructions in at least one of three domain areas of post-cesarean care: wound care, planning of future births, and postpartum depression. The studies did not need to have a primary focus of discharge instructions to be included, but they needed to have mention of instructions somewhere within the text. We defined discharge instructions as the overall set of instructions and actions provided to women following medically advised leave from the healthcare facility, and which can take the form of words, documentation, infographics, video content and can be delivered by any member of the healthcare team or as part of scientific research procedures. We defined wound care as instructions that captured concepts of antibiotic uses, surgical site infection and surgical care. Planning of future births was defined as instructions that encompassed services of contraception, family planning, repeat c-section, trial of labor after c-section and breastfeeding. Postpartum depression was defined as development of self-limited mild depressive symptoms or syndromes of minor and major depression within six weeks following delivery, which encapsulated concepts of depression, mental health, blues and counselling. At least two independent reviewers conducted full text review (JM, JS, AN, AU). Where the two reviewers disagreed, a third independent reviewer established eligibility. A detailed outline of studies included throughout the various stages of screening can be found in Fig 1.

### Stage 4: Data charting

The research team developed a data charting form that captured authors’ last name, publication year, study design, type of intervention, country of implementation, and instructions for future discharge instructions. Importantly, discharge instructions were extracted from various sections of the studies included, from passing comments in the method sections to practices implemented as part of a primary intervention.

### Stage 5: Collection, summary, reporting and quality assessment

**Fig 2:**
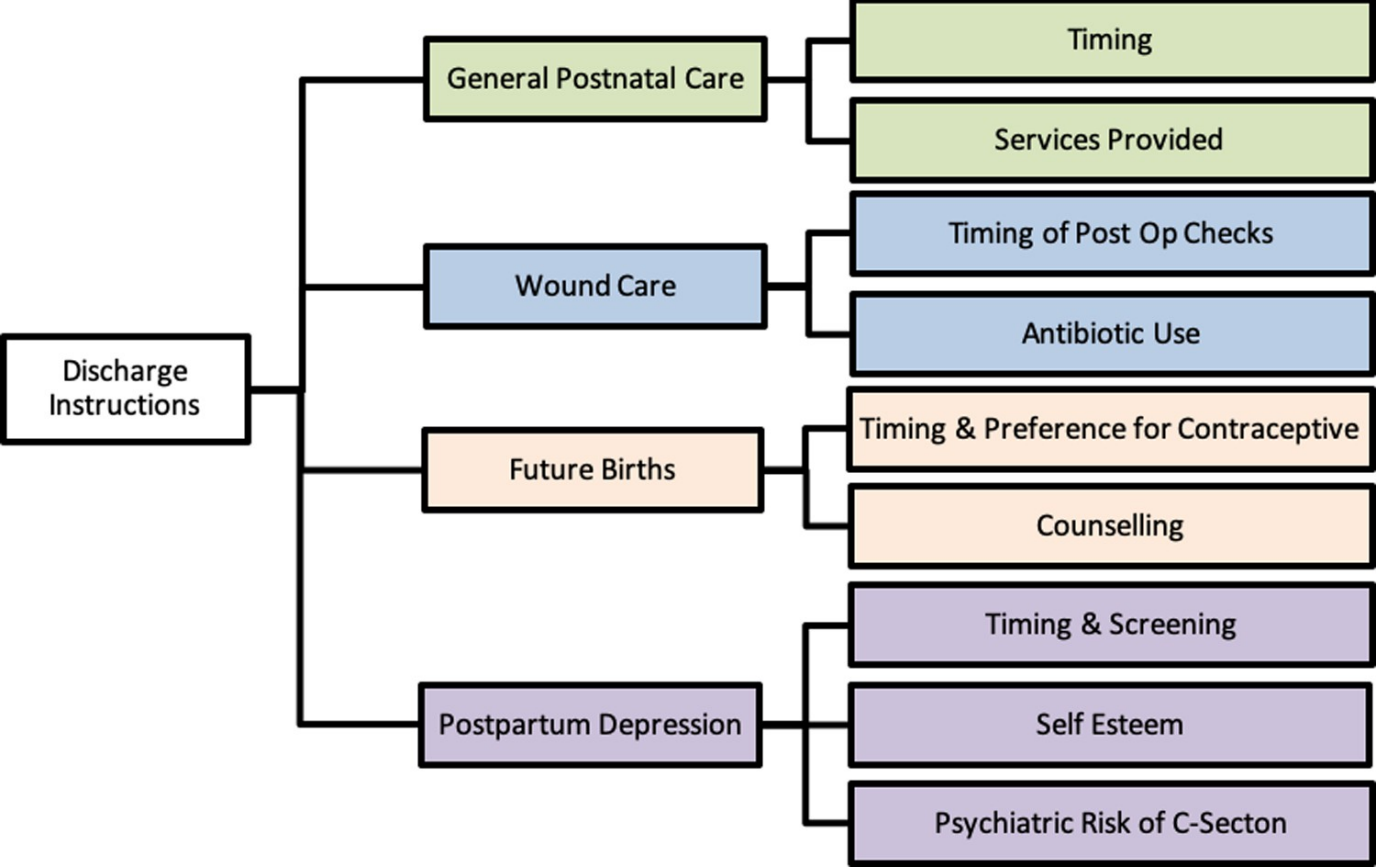
Thematic groups of data analysis.

Data from the charting form were organized into four major categories: general postnatal care, wound care, future births, and postpartum depression. For each of these categories, we determined nascent thematic groups (Fig 2) and reported trends within each theme using vote counting and narrative assessment. Results are reported under these four major categories. Assessment of study quality was performed using the GRADE system [20]. The full evaluation of grade ratings for the outcomes explored in this scoping review can be found in Table B in S1 Text.

## Results

We identified 6462 unique articles from our initial search, of which 78 studies met inclusion criteria for the scoping review. Of these 78 studies, 5 directly evaluated discharge protocols and 73 included information on discharge instructions in the context of a different study objective. Included publications began as early as 1993, with peak publication between 2012–2015, and again between 2017–2019. Studies were evenly distributed between rural and urban settings, with 37.6% of studies recording participants in an urban setting, 35.1% recording participants in a rural setting, 24.6% of studies recording participants in a suburban setting, and 2.6% of studies occurring in mixed urban, rural, and suburban settings. 60.2% of studies were carried out at public institutions, 6.4% at private institutions, and 33% in joint public-private institutions. Of the 78 studies included, 23.1% came from Nigeria, 17.7% from Ethiopia, and 12.8% from Tanzania. The remaining articles were distributed across south and central Africa. No documents from ministry of health websites or professional society websites were found that met criteria for inclusion.

## General postnatal care

Of the 78 studies included in this review, 16 studies contained instructions on general postnatal care. 5 of these addressed the range of services provided in the postnatal period, while 6 addressed timing of postnatal care. Among the studies on postnatal services, 4 of the 5 highlighted the need for breastfeeding counselling [21–24]. Separately, 1 study provided participants with a vaginal exam as part of the postnatal services rendered at the 6 week visit [25]. Regarding timing of general postnatal care, 2 studies identified the first 4–24 hours after delivery or immediate postpartum period as a prime time to provide postnatal services due to higher rates of attendance compared to later time points [26, 27], while 3 studies recommended patients receive a full check-in after six weeks [21, 26, 28]. Collectively the studies identified gaps in postnatal care, specifically the need for clearer protocols highlighting: what information needs to be shared at discharge, when an appropriate time for follow-up visits should be, what tasks need to be carried out at these visits, and who in the workforce should carry out these tasks.

## Wound care

There were a total of 37 studies in which participants were given instructions related to wound care, with recurring themes of postoperative evaluations and antibiotic use. Among the 37 studies, 18 provided instructions covering post-operative evaluation. These studies differed in timing of postoperative evaluation, tasks for evaluation, and mode of evaluation. Details on instructions covering post-evaluation check are described in Table 1. Out of the 37 studies on wound care, 16 studies reported providing patients with an antibiotic regimen, with 5 of these studies detailing the specific antibiotic course and duration [29–33]. Antibiotic use varied across studies with differing pre- and postoperative regimens as well as post-discharge regimens in some. Several studies identified key gaps in wound care management. One highlighted the need for clear protocols in determining what to assess at each postoperative follow-up, with the recommendation that a structured and validated questionnaire be developed and implemented broadly [34]. Lastly, a few studies suggested that future guidelines for wound care should identify and account for risk factors that are associated with an increased likelihood of developing surgical site infections [35, 36].

**Table 1:** Modalities of postoperative wound checks.

Timing of Post-Op Check	Tasks for Evaluation	Mode of Evaluation	Number of Studies
3 days	Dressing changes and wound inspection [31, 35–38].	In person.	5
7-days	Removal of skin sutures, inspection of surgical wounds for purulent discharge, changing wound dressing, measuring axillary temperature.	In person.	6
10-days	Evaluate for SSI symptoms and fever, with possible removal of sutures [38–40].	In person.	3
15 days	At discharge, patients told to return sooner if they develop fever, foul smelling vaginal discharge, discharge from wound margin, bleeding, and breast engorgement. Otherwise checkup at 2 weeks for SSI evaluation. 1 study had patients provided with at-home thermometer [31, 32, 39, 41–43].	All in person, with the exception of 1 study called patients for SSI evaluation over the phone [43].	5
30-days	At discharge, patients told to return sooner if SSI symptoms are developed. Otherwise, checkup at 30 days for SSI evaluation. 1 study called patient at 15-days post-op for SSI evaluation over the phone ahead of 30-day check-up [32, 33, 36, 42–44].	1 study called patients at 15- and 30-days post-op, with in person visits exclusively for those with SSI symptoms [43]. 1 study only called patients at 30 days post-op with no in person visits [44].	6
6-weeks	At discharge, patients told to return if they develop fever, wound dehiscence, foul smelling lochia. Otherwise, check up at 6 weeks [36, 45–47].	In1 study, patients who were found to have persistent pain at 6 weeks were called at 6- and 12-months after delivery to investigate pain [46].	4

## Future births

We found 19 studies detailing instructions on planning for future births. General themes amongst these were contraceptive use and counselling. Specific to contraception, 6 studies reported and encouraged patients to use intrauterine devices (IUDs) [48–53], with 1 study describing a government family planning program that provided a wider range of contraceptives that also included oral contraceptives, condoms and other barrier methods [49]. Of the 5 studies on IUDs, 3 encouraged placement of IUD immediately postpartum with the option of IUD placement at 6 weeks for those who declined immediate placement [50, 51, 54]. Two studies raised concerns about patients asking their husbands for permission to place IUD, leading to delayed placement [49, 54]. Regarding family planning counselling, 6 studies offered counselling services to meet the needs of c-section patients [55–60]. 1 study provided its participants with several hours of counseling after delivery, encouraging husbands to join in order to educate both parents on the importance of family planning in the aftermath of a c-section [57].

## Postpartum depression

Overall, there was a paucity of studies on postpartum depression as it related to c-section. Of the 78 studies included in the scoping review, only 5 studies contained information on discharge instructions around depression specific to the c-section patient population. Among these, three recurring themes were: timing of screening, self-esteem, and association between emergency c-sections and psychiatric morbidity. Concerning timing, one study recommended patients return to the hospital for screening at 6–8 weeks [61]. Another study demonstrated evidence of widespread major depressive disorder even following the screening period at 4–6 weeks, prompting suggestion of extended counselling services at three-months, six-months, and nine-months [41]. Regarding self-esteem, two studies broached the issues of esteem associated with c-sections [11, 13], with 1 highlighting that many women faced societal expectations of vaginal delivery and subsequently reported higher rates of postpartum depression at 6 weeks postpartum likely related to feelings of reduced pride and self-esteem [11]. However, neither study included specific guidelines on how to address this finding. Regarding the increased psychiatric risk with emergency c-sections, 3 studies collectively engaged with this theme. One study showed that women who had undergone life-saving c-sections were at increased risk of psychological distress, specifically pointing out that the fear of cesarean itself might be a vulnerability for the development of postnatal emotional distress [61]. A different study highlighted that this fear of emergency c-section was not exclusively due to expenses and the life threatening dangers associated with surgical delivery, but rather the risk of compromising future childbearing [58]. Another study found that emergency c-sections were associated with increased risk for post-traumatic stress disorder (PTSD) while elective c-sections were not, suggesting that unplanned operations rather than operative interventions themselves were a predictor of PTSD [13].

## Discussion

Our review sought to identify the different discharge instructions provided to women undergoing c-section on general postnatal care, wound care, future births, and postpartum depression. We found a broad range of studies that included some information on discharge instructions provided to women; however, few studies directly evaluated discharge instruction strategies. Regarding wound care, we found that recommendations for postoperative check-ups were highly variable in terms of timing and in terms of what these check-ups consisted of, with a clear need for well-defined protocols. For planning future births, instructions highlighted that contraception was often provided prior to discharge or at a close follow-up appointment, with important consideration given to the inclusion of husbands in counselling services. With regards to postpartum depression, our review found very few studies providing instructions specific to c-section patients. Of those identified, recurring themes were timing the postpartum screening to 4–8 weeks after discharge, and associations between c-section and self-esteem, and between c-section and psychiatric morbidity.

The findings in this review illustrate a broad range of discharge instructions for c-section patients. These instructions, and their subsequent impression on the postpartum landscape in sub-Saharan Africa, show a lack of standardized consensus and specificity for patients with unique circumstances. The instructions identified in this review stand in sharp contrast to those provided for antepartum care in sub-Saharan Africa, which are notable for their specificity, standardization and detail. Likewise, the instructions identified here are also distinguished from those of peripartum care, where surgical checklists provide step-by-step details on evaluating candidates for c-section and subsequently providing operative care [14–16]. While there remains ample room for antenatal protocols to provide education on wound care to women with an early indication for c-section as well as to prepare all patients for family planning and mental health care, the findings from this review establish a clear need for standardized consensus protocols on discharge instructions following c-section as part of a larger vision for postpartum care in sub-Saharan Africa. With a standard set of consensus protocols as a framework, individual countries and regions within sub-Saharan Africa could build more specific protocols adapted to their local context.

Furthermore, for the provision of discharge instructions to become an effective practice, they need to account for several limiting factors unique to the sub-Saharan setting. One of these is the financial burden to follow-up, where hospital fees and cost of transport often risk pushing patients and their families into financial catastrophe [29, 58, 62]. Another limiting factor is the lack of access to physical materials such as bandages, clean water, and feminine hygiene products, all of which can limit individual capacity to follow through on instructions provided [18, 63]. Importantly, it is paramount that instructions be written in clear and cogent language in order to be accessible to patients with varying levels of literacy [53, 64]. Lastly, the lack of consistency in discharge instructions runs the risk of leaving patients confused on how to navigate existing healthcare infrastructure, even where services are available [65]. Without standardized consensus protocols for discharge instructions, we risk sending patients home without sufficient supports to ensure their safety and recovery.

### Strengths and limitations

Our study used a broad search strategy that included sources from published manuscripts and documents from ministry of health websites, professional organization websites, and other grey literature sources. Additionally, our study included both English and French language papers which expanded the scope of potential search results and our ability to find relevant material. For each of the sources identified, the screening process involved full evaluation and data extraction of instructions from all sections of each source, not just extraction of primary outcomes.

One of the limitations of this study was the paucity of literature available. None of the documents reviewed from ministry of health websites or professional society websites met criteria for inclusion. On specific topics such as discharge instructions related to postpartum depression, articles were few and challenging to find. Additionally, given the heterogenous nature of the studies and the fact that many of these instructions were found in different sections of the respective papers, we were unable to perform quantitative review of outcomes. Importantly, while our review highlights the instructions provided to patients, the impact of those instructions to clinical outcomes and patient well-being have yet to be evaluated.

## Conclusions

There is a need to develop clear consensus protocols for the discharge instructions that women receive following c-section. These protocols should address the clinical needs of women as they relate to timing of follow-up appointments and the required services provided at these appointments for wound care, planning of future births, and postpartum depression. Importantly, future instructions should account for financial burden of hospitalization, access to the physical resources necessary for postpartum care, and education of patients and communities to reduce the stigma often associated with c-sections.

## Supporting information

S1 Checklist Reporting items for systematic & scoping review. (DOCX)

S1 Text (DOCX)
