# Supplementary material for: Discharge instructions given to women following delivery by cesarean section in Sub-Saharan Africa: A scoping review
Source: PLOS Glob Public Health. 2022 Apr 22;2(4):e0000318. doi: 10.1371/journal.pgph.0000318 (PMC10021225; doi:10.1371/journal.pgph.0000318)
Supplement: S1 Text — (DOCX) [file pgph.0000318.s002.docx]

**Table A: Databases, Search Terms, & Studies Recorded**

| **Source** | **Search terms** | **# records** |
| --- | --- | --- |
| PubMed | ((C-section[Title/Abstract]) OR (cesarean[Title/Abstract]) OR (caesarean[Title/Abstract]) OR (cesarean section[Title/Abstract]) OR (caesarean section[Title/Abstract]) OR (abdominal delivery[Title/Abstract]) OR (surgical birth[Title/Abstract]) OR (surgical delivery[Title/Abstract]) OR (C-section[MeSH Terms]) OR (cesarean[MeSH Terms]) OR (caesarean[MeSH Terms]) OR (cesarean section[MeSH Terms]) OR (caesarean section[MeSH Terms]) OR (abdominal delivery[MeSH Terms]) OR (surgical birth[MeSH Terms]) OR (surgical delivery[MeSH Terms])) AND ((Sub-Sahara[Title/Abstract]) OR (Sub-Saharan[Title/Abstract]) OR (Angola[Title/Abstract]) OR (Benin[Title/Abstract]) OR (Botswana[Title/Abstract]) OR (Burkina Faso[Title/Abstract]) OR (Burundi[Title/Abstract]) OR (Cameroon[Title/Abstract]) OR (Cape Verde[Title/Abstract]) OR (Central African Republic[Title/Abstract]) OR (Chad[Title/Abstract]) OR (Comoros[Title/Abstract]) OR (Congo Brazzaville[Title/Abstract]) OR (Congo[Title/Abstract]) OR (Democratic Republic of the Congo[Title/Abstract]) OR (Cote d'Ivoire[Title/Abstract]) OR (Djibouti[Title/Abstract]) OR (Equatorial Guinea[Title/Abstract]) OR (Eritrea[Title/Abstract]) OR (Ethiopia[Title/Abstract]) OR (Gabon[Title/Abstract]) OR (Gambia[Title/Abstract]) OR (Ghana[Title/Abstract]) OR (Guinea[Title/Abstract]) OR (Guinea Bissau[Title/Abstract]) OR (Kenya[Title/Abstract]) OR (Lesotho[Title/Abstract]) OR (Liberia[Title/Abstract]) OR (Madagascar[Title/Abstract]) OR (Malawi[Title/Abstract]) OR (Mali[Title/Abstract]) OR (Mauritania[Title/Abstract]) OR (Mauritius[Title/Abstract]) OR (Mozambique[Title/Abstract]) OR (Namibia[Title/Abstract]) OR (Niger[Title/Abstract]) OR (Nigeria[Title/Abstract]) OR (Réunion[Title/Abstract]) OR (Rwanda[Title/Abstract]) OR (Sao Tome[Title/Abstract] AND Principe[Title/Abstract]) OR (Senegal[Title/Abstract]) OR (Seychelles[Title/Abstract]) OR (Sierra Leone[Title/Abstract]) OR (Somalia[Title/Abstract]) OR (South Africa[Title/Abstract]) OR (Sudan[Title/Abstract]) OR (Swaziland[Title/Abstract]) OR (Tanzania[Title/Abstract]) OR (Togo[Title/Abstract]) OR (Uganda[Title/Abstract]) OR (Western Sahara[Title/Abstract]) OR (Zambia[Title/Abstract]) OR (Zimbabwe[Title/Abstract]) OR (Sub-Sahara[MeSH Terms]) OR (Sub-Saharan[MeSH Terms]) OR (Angola[MeSH Terms]) OR (Benin[MeSH Terms]) OR (Botswana[MeSH Terms]) OR (Burkina Faso[MeSH Terms]) OR (Burundi[MeSH Terms]) OR (Cameroon[MeSH Terms]) OR (Cape Verde[MeSH Terms]) OR (Central African Republic[MeSH Terms]) OR (Chad[MeSH Terms]) OR (Comoros[MeSH Terms]) OR (Congo Brazzaville[MeSH Terms]) OR (Congo[MeSH Terms]) OR (Democratic Republic of the Congo[MeSH Terms]) OR (Cote d'Ivoire[MeSH Terms]) OR (Djibouti[MeSH Terms]) OR (Equatorial Guinea[MeSH Terms]) OR (Eritrea[MeSH Terms]) OR (Ethiopia[MeSH Terms]) OR (Gabon[MeSH Terms]) OR (Gambia[MeSH Terms]) OR (Ghana[MeSH Terms]) OR (Guinea[MeSH Terms]) OR (Guinea Bissau[MeSH Terms]) OR (Kenya[MeSH Terms]) OR (Lesotho[MeSH Terms]) OR (Liberia[MeSH Terms]) OR (Madagascar[MeSH Terms]) OR (Malawi[MeSH Terms]) OR (Mali[MeSH Terms]) OR (Mauritania[MeSH Terms]) OR (Mauritius[MeSH Terms]) OR (Mozambique[MeSH Terms]) OR (Namibia[MeSH Terms]) OR (Niger[MeSH Terms]) OR (Nigeria[MeSH Terms]) OR (Réunion[MeSH Terms]) OR (Rwanda[MeSH Terms]) OR (Sao Tome[MeSH Terms] AND Principe[MeSH Terms]) OR (Senegal[MeSH Terms]) OR (Seychelles[MeSH Terms]) OR (Sierra Leone[MeSH Terms]) OR (Somalia[MeSH Terms]) OR (South Africa[MeSH Terms]) OR (Sudan[MeSH Terms]) OR (Swaziland[MeSH Terms]) OR (Tanzania[MeSH Terms]) OR (Togo[MeSH Terms]) OR (Uganda[MeSH Terms]) OR (Western Sahara[MeSH Terms]) OR (Zambia[MeSH Terms]) OR (Zimbabwe[MeSH Terms])) | 3888 |
| Globus Index Medicus | (tw:((tw:(C-section)) OR (tw:(cesarean section)) OR (tw:(caesarian section)) OR (tw:(cesarean )) OR (tw:(caesarean)) OR (tw:(abdominal delivery)) OR (tw:(surgical birth)) OR (tw:(surgical delivery)))) AND (tw:((tw:(sub-sahara)) OR (tw:(sub-saharan)) OR (tw:(angola)) OR (tw:(benin)) OR (tw:(botswana)) OR (tw:(burkina faso)) OR (tw:(burundi)) OR (tw:(cameroon)) OR (tw:(cape verde)) OR (tw:(central african republic)) OR (tw:(chad)) OR (tw:(comoros)) OR (tw:(congo brazzaville)) OR (tw:(democratic republic of the congo)) OR (tw:(cote d'ivoire)) OR (tw:(djibouti)) OR (tw:(equatorial guinea)) OR (tw:(eritrea)) OR (tw:(ethiopia)) OR (tw:(gabon)) OR (tw:(gambia)) OR (tw:(ghana)) OR (tw:(guinea)) OR (tw:(guinea bissau)) OR (tw:(kenya)) OR (tw:(lesotho)) OR (tw:(liberia)) OR (tw:(madagascar)) OR (tw:(malawi)) OR (tw:(mali)) OR (tw:(mauritania)) OR (tw:(mauritius)) OR (tw:(mozambique)) OR (tw:(namibia)) OR (tw:(niger)) OR (tw:(nigeria)) OR (tw:(reunion)) OR (tw:(rwanda)) OR (tw:(sao tome and principe)) OR (tw:(senegal)) OR (tw:(seychelles)) OR (tw:(sierra leone)) OR (tw:(somalia)) OR (tw:(south africa)) OR (tw:(sudan)) OR (tw:(swaziland)) OR (tw:(tanzania)) OR (tw:(togo)) OR (tw:(uganda)) OR (tw:(western sahara)) OR (tw:(zambia)) OR (tw:(zimbabwe)))) | 270 |
| NiPAD (Africa-Wide) | TX ( C-section or Cesarean Section or Caesarean Section or Caesarean or Cesarean or Abdominal Delivery or Surgical Birth or Surgical Delivery ) AND TX ( sub-sahara OR sub-saharan OR angola OR benin OR botswana OR burkina faso OR burundi OR cameroon OR cape verde OR central african republic OR chad OR comoros OR congo brazzaville OR democratic republic of the congo OR cote d'ivoire OR djibouti OR equatorial guinea OR eritrea OR ethiopia OR gabon OR gambia OR ghana OR guinea OR guinea bissau OR kenya OR lesotho OR liberia OR madagascar OR malawi OR mali OR mauritania OR mauritius OR mozambique OR namibia OR niger OR nigeria OR reunion OR rwanda OR sao tome and principe OR senegal OR seychelles OR sierra leone OR somalia OR south africa OR sudan OR swaziland OR tanzania OR togo OR uganda OR OR western sahara OR zambia OR zimbabwe | 2649 |
| EMBASE | ('c section'/exp OR 'cesarean section'/exp OR 'caesarean section'/exp OR caesarean OR cesarean OR 'abdominal delivery' OR 'surgical birth' OR 'surgical delivery'/exp OR 'c section':ti,ab,kw OR 'cesarean section':ti,ab,kw OR 'caesarean section':ti,ab,kw OR caesarean:ti,ab,kw OR cesarean:ti,ab,kw OR 'abdominal delivery':ti,ab,kw OR 'surgical birth':ti,ab,kw OR 'surgical delivery':ti,ab,kw) AND ('sub sahara' OR 'sub saharan' OR 'angola'/exp OR 'benin'/exp OR 'botswana'/exp OR 'burkina faso'/exp OR 'burundi'/exp OR 'cameroon'/exp OR 'cape verde'/exp OR 'central african republic'/exp OR 'chad'/exp OR 'comoros'/exp OR 'congo brazzaville'/exp OR 'democratic republic of the congo'/exp OR 'cote divoire' OR 'djibouti'/exp OR 'equatorial guinea'/exp OR 'eritrea'/exp OR 'ethiopia'/exp OR 'gabon'/exp OR 'gambia'/exp OR 'ghana'/exp OR 'guinea'/exp OR 'guinea bissau'/exp OR 'kenya'/exp OR 'lesotho'/exp OR 'liberia'/exp OR 'madagascar'/exp OR 'malawi'/exp OR 'mali'/exp OR 'mauritania'/exp OR 'mauritius'/exp OR 'mozambique'/exp OR 'namibia'/exp OR 'niger'/exp OR 'nigeria'/exp OR 'reunion'/exp OR 'rwanda'/exp OR 'sao tome and principe'/exp OR 'senegal'/exp OR 'seychelles'/exp OR 'sierra leone'/exp OR 'somalia'/exp OR 'south africa'/exp OR 'sudan'/exp OR 'swaziland'/exp OR 'tanzania'/exp OR 'togo'/exp OR 'uganda'/exp OR 'western sahara'/exp OR 'zambia'/exp OR 'zimbabwe'/exp OR 'sub sahara':ti,ab,kw OR 'sub saharan':ti,ab,kw OR angola:ti,ab,kw OR benin:ti,ab,kw OR botswana:ti,ab,kw OR 'burkina faso':ti,ab,kw OR burundi:ti,ab,kw OR cameroon:ti,ab,kw OR 'cape verde':ti,ab,kw OR 'central african republic':ti,ab,kw OR chad:ti,ab,kw OR comoros:ti,ab,kw OR 'congo brazzaville':ti,ab,kw OR 'democratic republic of the congo':ti,ab,kw OR 'cote divoire':ti,ab,kw OR djibouti:ti,ab,kw OR 'equatorial guinea':ti,ab,kw OR eritrea:ti,ab,kw OR ethiopia:ti,ab,kw OR gabon:ti,ab,kw OR gambia:ti,ab,kw OR ghana:ti,ab,kw OR guinea:ti,ab,kw OR 'guinea bissau':ti,ab,kw OR kenya:ti,ab,kw OR lesotho:ti,ab,kw OR liberia:ti,ab,kw OR madagascar:ti,ab,kw OR malawi:ti,ab,kw OR mali:ti,ab,kw OR mauritania:ti,ab,kw OR mauritius:ti,ab,kw OR mozambique:ti,ab,kw OR namibia:ti,ab,kw OR niger:ti,ab,kw OR nigeria:ti,ab,kw OR reunion:ti,ab,kw OR rwanda:ti,ab,kw OR 'sao tome and principe':ti,ab,kw OR senegal:ti,ab,kw OR seychelles:ti,ab,kw OR 'sierra leone':ti,ab,kw OR somalia:ti,ab,kw OR 'south africa':ti,ab,kw OR sudan:ti,ab,kw OR swaziland:ti,ab,kw OR tanzania:ti,ab,kw OR togo:ti,ab,kw OR uganda:ti,ab,kw OR 'western sahara':ti,ab,kw OR zambia:ti,ab,kw OR zimbabwe:ti,ab,kw) | 3117 |
| Global Health (EBSCO) | TX ( C-section or Cesarean Section or Caesarean Section or Caesarean or Cesarean or Abdominal Delivery or Surgical Birth or Surgical Delivery ) AND TX ( sub-sahara OR sub-saharan OR angola OR benin OR botswana OR burkina faso OR burundi OR cameroon OR cape verde OR central african republic OR chad OR comoros OR congo brazzaville OR democratic republic of the congo OR cote d'ivoire OR djibouti OR equatorial guinea OR eritrea OR ethiopia OR gabon OR gambia OR ghana OR guinea OR guinea bissau OR kenya OR lesotho OR liberia OR madagascar OR malawi OR mali OR mauritania OR mauritius OR mozambique OR namibia OR niger OR nigeria OR reunion OR rwanda OR sao tome and principe OR senegal OR seychelles OR sierra leone OR somalia OR south africa OR sudan OR swaziland OR tanzania OR togo OR uganda OR western sahara OR zambia OR zimbabwe ) | 61 |

Searched on August 5, 2020

**Table B: GRADE Approach of Evidence Quality**

| **№ of studies** | **Certainty assessment** | | | | | | **impact** | **Certainty** |
| --- | --- | --- | --- | --- | --- | --- | --- | --- |
|  | **Study design** | **Risk of bias** | **Inconsistency** | **Indirectness** | **Imprecision** | **Other considerations** |  |  |
| General Post Natal Care – Services Provided (assessed with: services provided) | | | | | | | | |
| 5 | Observational studies | not serious | not serious | not serious | serious ^a^ | none | Quality of evidence for this outcome was moderate, limited by imprecision as studies reported a broad range of services, rather than particular focus on a few key services. | ⨁⨁⨁◯ MODERATE |
| General Post Natal Care – Timing (assessed with: days after delivery) | | | | | | | | |
| 6 | Systematic Reviews & Observational Studies | not serious | not serious | serious ^b^ | not serious | none | Quality of evidence for this outcome to be moderate primarily due to indirectness, as most studies included were not primarily focused on timing as a metric. | ⨁⨁⨁◯ MODERATE |
| Wound Care – Timing of Post-Op Check (assessed with: days or weeks after c-section) | | | | | | | | |
| 18 | Randomized Control Trials and Cohort Studies | not serious | not serious | not serious | not serious | strong association | The quality of evidence for timing of post-op checks as an outcome was found to be high, with good precision around SSIs and strong grounding evidence. | ⨁⨁⨁⨁ HIGH |
| Wound Care – Antibiotic Regimen (assessed with: course & duration of antibiotic use) | | | | | | | | |
| 5 | Observational Studies and  Randomized Control Trials | not serious | not serious | not serious | not serious | strong association | Quality of evidence was high, as a majority of studies were primarily focused on antibiotic use, hence high levels of directness. Additionally, these studies largely recommended antibiotic use overall, showing precision. | ⨁⨁⨁⨁ HIGH |
| Future Births – Contraceptive Use | | | | | | | | |
| 6 | Randomized Control Trials, Systematic Reviews, Cohort Studies | not serious | not serious | not serious | serious ^c^ | none | Quality of evidence for this outcome was found to be moderate, limited by imprecision as studies did not show commonality around primary focus. | ⨁⨁⨁◯ MODERATE |
| Future Births – Counselling Services | | | | | | | | |
| 6 | Randomized Control Trials, Systematic Reviews, Cohort Studies | not serious | not serious | serious ^d^ | serious ^e^ | none | Quality of evidence for this outcome was found to be low, limited by indirectness and imprecision, as studies included were not primarily focused on the outcome of counselling and where counselling was provided, it took many forms. | ⨁⨁◯◯ LOW |

a. There were only 5 studies included here, and each discussed distinct service provision, with only two overlapping services: breast feeding information and teaching of postnatal danger signs.

b. None of the 6 studies included has as a primary goal measuring the best timing for postnatal care, but rather commented on timing based on observations

c. Very few studies overall, and while there were thematic similarities, studies were mostly unrelated.

d. Only 2 of the 6 studies were primarily focused on counseling. The remaining 4 offered up remarks on their counseling practices.

e. Studies were mostly unrelated, with shared thematic similarities.

**Table C: Characterization of Studies Included in Scoping Review**

| **Title of Article** | **First Author, Last Name** | **Year of Publication** | **Journal Name** | **Name of Country** | **Geographical Setting** | **Healthcare Leadership** |
| --- | --- | --- | --- | --- | --- | --- |
| The use of antenatal and postnatal care: perspectives and experiences of women and health care providers in rural southern Tanzania | Mrisho | 2009 | BMC Pregnancy & Childbirth | Tanzania | Rural | Public |
| Incidence and risk factors for caesarean wound infection in Lagos, Nigeria | Ezechi | 2009 | BioMed Central | Lagos, Nigeria | Urban | Private |
| Involving male partners in maternity care in Burkina Faso: a randomized controlled trial | Daniele | 2018 | Bulletin of the World Health Organization | Burkina Faso | Urban | Public & Private |
| Experiences of Mothers with Antenatal, Delivery and Postpartum Care in Rural Gambia | Telfer | 2002 | African Journal of Reproductive Health | Gambia | Rural | Public |
| Impact of guidelines implementation for the rational use of prophylactic antibiotics in elective cesarean sections at Elqutainah Teaching Hospital | Suliman | 2020 | Journal of Family Medicine and Primary Care | Sudan | Urban | Private |
| Cost Implication of Abdominal wound Dehiscence in Obstetric Patients at UNTH Enugu, 1990 - 1999 | Aniebue | 2003 | Journal of College of Medicine | Nigeria | Urban | Public |
| Association between skilled maternal healthcare and postpartum contraceptive use in Ethiopia | Tessema | 2018 | BMC Pregnancy and Childbirth | Ethiopia | Rural | Public & Private |
| Women's perceptions of antenatal, delivery, and postpartum services in rural Tanzania | Mahiti | 2015 | Global Health Action | Tanzania | Rural | Public |
| Vaginal Birth After Caesarean Section in Low Resource Settings: The Clinical and Ethical Dilemma | Wanyonyi | 2015 | Journal of Obstetrics and Gynaecology | Kenya | Suburban | Public & Private |
| Persistent pain after caesarean and vaginal delivery: Experience at a public tertiary hospital, Benin city, Nigeria | Enaruna | 2019 | Tropical Journal of Obstetrics and Gynaecology | Nigeria | Urban | Public |
| Women's experiences of midwifery care immediately before and after caesarean section deliveries at a publical hospital in the Western Region of Ghana | Afaya | 2020 | BMC Pregnancy and Childbirth | Ghana | Rural | Public |
| Stage of labor at admission among Ugandan women with a prior cesarean, and its impact on management and delivery outcomes | Boatin | 2017 | International Journal of Gynecology & Obstetrics | Uganda | Urban | Public |
| Prevalence and root causes of surgical site infection among women undergoing caesarean section in Ethiopia: a systematic review and meta-analysis | Adane | 2019 | Patient Safety in Surgery | Ethiopia | Suburban | Public & Private |
| Family planning and desire for additional children after cesarean section | Maier | 1993 | International Journal of Gynecology & Obstetrics | Burkina Faso | Rural | Public |
| Prevalence and predictors of surgical-site infection after caesarean section at a rural district hospital in Rwanda | Nkurunziza | 2019 | The British journal of surgery | Rwanda | Rural | Public |
| After surgery: the effects of life-saving caesarean sections in Burkina Faso | Filippi | 2015 | BMC Pregnancy and Childbirth | Burkina Faso | Suburban | Public & Private |
| Saving Mothers, Giving Life: It Takes a System to Save a Mother | Conlon | 2019 | Global Health: Science and Practice | Uganda and Zambia | Rural | Public & Private |
| Access to facility delivery and caesarean section in north-central Liberia: a cross sectional community-based study | Gartland | 2012 | BMJ | Liberia | Rural | Public |
| Incidence of Surgical Site Infection and Factors Associated among Cesarean Deliveries in Selected Government Hospitals in Addis Ababa, Ethiopia, 2019 | Lijaemiro | 2020 | Obstetrics and Gynecology International | Ethiopia | Urban | Public |
| A one-year cohort study of complications, continuation, and failure rates of postpartum TCu380A in Tanzania | Rwegoshora | 2020 | Reproductive Health | Tanzania | Suburban | Public |
| Caesarean section and subsequent fertility in sub-Saharan Africa | Collin | 2006 | BJOG : an international journal of obstetrics and gynaecology | 22 SSA Countries | Suburban | Public & Private |
| Post-caesarean section surgical site infections at a Tanzanian tertiary hospital: a prospective observational study | De Nardo | 2016 | Journal of Hospital Infection | Tanzania | Urban | Public |
| A randomised controlled trial of uterine exteriorisation and non-exteriorisation at caesarean section | Orji | 2008 | Australian and New Zealand Journal of Obstetrics and Gynaecology | Nigeria | Rural | Public |
| A national review of cesarean delivery in Ethiopia | Fesseha | 2011 | International Journal of Gynecology and Obstetrics | Ethiopia | Suburban | Public & Private |
| Incidence and predictors of surgical site infections following caesarean sections at Bugando Medical Centre, Mwanza, Tanzania | Mpogoro | 2014 | Antimicrobial Resistance and Infection Control | Tanzania | Urban | Public & Private |
| Surgical site infection and associated factors among women underwent cesarean delivery in Debretabor General Hospital, Northwest Ethiopia: hospital based cross sectional stud | Molla | 2019 | BMC Pregnancy and Childbirth | Ethiopia | Rural | Public |
| Postoperative outcome of caesarean sections and other major emergency obstetric surgery by clinical officers and medical officers in Malawi | Chilopora | 2007 | Human Resources for Health | Malawi | Suburban | Public |
| Caesarean section deliveries: Experiences of mothers of midwifery care at a public hospital in Nelson Mandela Bay | Jikijela | 2018 | Curationis | South Africa | Urban | Public |
| Uncomplicated Caesarian section: Is prolonged hospital stay necessary? | Fasubaa | 2000 | East African Medical journal | Nigeria | Rural | Public |
| Predictors of exclusive breastfeeding duration among 6-12 month aged children in gurage zone, South Ethiopia: a survival analysis | Kasahun | 2017 | International Breast Feeding Journal | Ethiopia | Suburban | Public & Private |
| Choice of antibiotics for infection prophylaxis in emergency cesarean sections in low-income countries: A cost-benefit study in Mozambique | Kayihura | 2003 | Acta Obstetricia et Gynecologica Scandinavica | Mozambique | Urban | Private |
| Cesarean section surgical site infections in sub-Saharan Africa: a multi-country study from Medecins Sans Frontieres | Chu | 2014 | World Journal of Surgery | Burundi, DRC, and Sierra Leone | Suburban | Private |
| Determinants of postnatal care utilization in Tigray, Northern Ethiopia: A community based cross-sectional study | Berhe | 2019 | PLOS ONE | Ethiopia | Urban or Suburban | Public & Private |
| What can we learn about postnatal care in Ghana if we ask the right questions? A qualitative study | Hill | 2015 | Global Health Action | Ghana | Suburban | Public & Private |
| Implementing a combined infection prevention and control with antimicrobial stewardship joint program to prevent caesarean section surgical site infections and antimicrobial resistance: a Tanzanian tertiary hospital experience | Gentilotti | 2020 | BMC Antimicrobial Resistance and Infection Control | Tanzania | Urban | Public |
| Fear, guilt, and debt: an exploration of women's experience and perception of cesarean birth in Burkina Faso, West Africa | Richard | 2014 | International Journal of Women's Health | Burkina Faso | Rural | Public & Private |
| Predictors for Persistent Perioperative Symptoms after a Cesarean Section at a Hospital in Rural Rwanda | Mukantwari | 2020 | Journal of the American College of Surgeons | Rwanda | Rural | Public |
| Intracesarean insertion of the Copper T380A versus 6 weeks postcesarean: a randomized clinical trial | Lester | 2015 | Contraception | Uganda | Urban | Public |
| [Vaginal birth after previous cesarean section in low-resource countries: healthcare chain and materno-fetal follow-up] | Koh | 2018 | Pan African Medical Journal | Cameroun | Urban | Public |
| Determinants of antenatal care, institutional delivery and postnatal care services utilization in Nigeria | Oche | 2015 | Pan African Medical Journal | Nigeria | Suburban | Public & Private |
| Determinants of early initiation of breastfeeding in rural Tanzania | Exavery | 2015 | International Breast Feeding Journal | Tanzania | Rural | Public & Private |
| Provision of postpartum care to women giving birth in health facilities in sub-Saharan Africa: A cross-sectional study using Demographic and Health Survey data from 33 countries | Benova | 2019 | PLOS Medicine | 33 SSA Countries | Suburban | Public & Private |
| Surgical site infection and its associated factors following cesarean section in Ethiopia: a cross-sectional study | Azeze | 2019 | BCM Research Notes | Ethiopia | Rural | Public |
| Surgical-site Infection Following Cesarean Section in Kano, Nigeria | Jido | 2012 | Annals of Medical and Health Sciences Research | Nigeria | Suburban | Public & Private |
| Post-traumatic stress disorder after childbirth in Nigerian women: prevalence and risk factors | Adewuya | 2005 | BJOG An International Journal of Obstetrics and Gynaecology | Nigeria | Rural | Public |
| Early Initiation and Exclusivity of Breastfeeding in Rural Zimbabwe: Impact of a Breastfeeding Intervention Delivered by Village Health Workers | Mbuya | 2019 | Maternal and Pediatric Nutrition | Zimbabwe | Rural | Public & Private |
| A randomised controlled trial of antibiotic prophylaxis in elective caesarean delivery | Bagratee | 2001 | British Journal of Obstetrics and Gynaecology | South Africa | Urban | Public |
| "You should go so that others can come"; the role of facilities in determining an early departure after childbirth in Morogoro Region, Tanzania | McMahon | 2015 | BMC Pregnancy and Childbirt | Tanzania | Suburban | Public & Private |
| Availability, utilisation and quality of maternal and neonatal health care services in Karamoja region, Uganda: a health facility-based survey | Wilunda | 2015 | Reproductive Health | Uganda | Rural | Public |
| Determinants of Contraception among Women with a Previous Ceasarean Section in the Kumasi Metropolis, Ghana | Otupiri | 2017 | Annals of Global Health | Ghana | Urban | Public |
| Prophylactic ampicillin versus cefazolin for the prevention of post-cesarean infectious morbidity in Rwanda | Mivumbi | 2014 | International Journal of Gynecology and Obstetrics | Rwanda | Urban | Public |
| Magnitude and determinants of surgical site infection among women underwent cesarean section in Ayder comprehensive specialized hospital Mekelle City, Tigray region, Northern Ethiopia, 2016 | Wendmagegn | 2018 | BMC Pregnancy and Childbirth | Ethiopia | Rural | Public |
| Postpartum emotional distress: a controlled study of Nigerian women after caesarean childbirth | Ukpong | 2006 | Journal of Obstetrics and Gynecology | Nigeria | Urban | Public |
| Cesarean section in relation to self-esteem and parenting among new mothers in southwestern Nigeria | Loto | 2010 | Acta Obstetricia et Gynecologica | Nigeria | Urban | Public |
| Sterilisation during unplanned caesarean sections for women likely to have a completed family-should they be offered? Experience in a country with limited health resources | Verkuyl | 2002 | BJOG: an International Journal of Obstetrics and Gynaecology | Zimbabwe | Suburban | Public |
| A Randomized Study Comparing Skin Staples with Subcuticular Sutures for Wound Closure at Caesarean Section in Black-Skinned Women | Abdus-Salam | 2014 | International Scholarly Research Notices | Nigeria | Urban | Public |
| A facility birth can be the time to start family planning: Postpartum intrauterine device experiences from six countries | Pfitzer | 2015 | International Journal of Gynecology and Obstetrics | Ethiopia and Rwanda | Suburban | Private |
| Post-operative Management in Uncomplicated Caesarean Delivery: A Randomised Trial of Short-Stay versus Traditional Protocol at the Lagos University Teaching Hospital, Nigeria | Oyeyemi | 2019 | Nigerian Postgraduate Medical Journal | Nigeria | Urban | Public |
| Caesarean sections in rural Burundi: how well are mothers doing two years on? | van den Boogaard | 2016 | Public Health Action | Burundi | Rural | Public & Private |
| Reliability and validity of using telephone calls for post-discharge surveillance of surgical site infection following caesarean section at a tertiary hospital in Tanzania | Nguhuni | 2017 | Antimicrobial Resistance and Infection Control | Tanzania | Suburban | Public & Private |
| Single-dose compared with multiple day antibiotic prophylaxis for cesarean section in low-resource settings, a randomized controlled, noninferiority trial | Westen | 2014 | Acta Obstetricia et Gynecologica Scandinavica | Tanzania | Rural | Public |
| Maternal morbidity in the first year after childbirth in Mombasa Kenya; a needs assessment | Chersich | 2009 | BMC Pregnancy and Childbirth | Kenya | Rural | Public |
| Postnatal Care Utilization and Associated Factors among Married Women in Benchi-Maji Zone, Southwest Ethiopia: A Community Based Cross-Sectional Study | Abota | 2018 | Ethop J Health Sci. | Ethiopia | Rural | Public |
| Determinants of Post-Caesarean Wound Infection at The University College Hospital Ibadan Nigeria | Morhason-Bello | 2009 | Nigerian Journal of Clinical Practice | Nigeria | Urban | Public |
| Availability and components of maternity services according to providers and users perspectives in North Gondar, northwest Ethiopia | Worku | 2013 | Reproductive Health | Ethiopia | Suburban | Public & Private |
| Prevalence and Determinants of Complete Postnatal Care Service Utilization in Northern Shoa, Ethiopia | Akibu | 2018 | Journal of Pregnancy | Ethiopia | Suburban | Public & Private |
| Feasibility, acceptability, and uptake of postpartum intrauterine contraceptive devices in southern Nigeria | Sodje | 2016 | International Journal of Gynecology and Obstetrics | Nigeria | Rural | Public & Private |
| The Efficacy of Two Doses versus 7 Days' Course of Prophylactic Antibiotics Following Cesarean Section: An Experience from Aminu Kano Teaching Hospital | Mohammed | 2020 | Annals of African Medicine | Nigeria | Urban | Public |
| The Effects of a Locally Developed mHealth Intervention on Delivery and Postnatal Care Utilization; A Prospective Controlled Evaluation among Health Centres in Ethiopia | Shiferaw | 2016 | Plos One | Ethiopia | Urban | Public |
| Quality cesarean delivery in Ouagadougou, Burkina Faso: A comprehensive approach | Richard | 2008 | Averting Maternal Death and Disability | Burkina Faso | Rural | Public |
| Assessment of discharge after 24 hours following elective caesarean section in Omdurman Maternity Hospital, Sudan, 2010 | Umbeli | 2012 | Sudan Journal of Medical Sciences | Sudan | Urban | Public |
| Bacteriology of Post Caesarean Wound Infection in a Specialist Hospital in Kano, North Western Nigeria | Shuaibu | 2013 | Sierra Leone Journal of Biomedical Research | Nigeria | Urban | Public |
| Risk Factors and Antibiogram of Organisms Causing Puerperal Sepsis in Tertiary Health Facilities in Nigeria | Ononuju | 2015 | Tropical Journal of Obstetrics & Gynecology | Nigeria | Urban | Public |
| Risk factors and microbial isolates of puerperal sepsis at the University of Maiduguri Teaching Hospital, Maiduguri, North-eastern Nigeria | Bako | 2012 | Archives of Gynecology & Obstetrics | Nigeria | Urban | Public |
| Quality of maternity care for adolescent mothers in Mbabane, Swaziland | Mngadi | 2002 | International Nursing Review | Swaziland | Urban | Public |
| Influences of health facility type for delivery and experience of cesarean section on maternal and newborn postnatal care between birth and facility discharge in Malawi | Kim | 2020 | BMC Health Services Research | Malawi | Rural, Urban, Suburban | Public & Private |
| Early feeding practices and associated  factors in Sudan: a cross-sectional analysis  from multiple Indicator cluster survey | Abdel-Rahman | 2020 | BMC International Breastfeeding Journal | Sudan | Rural, Urban, Suburban | Public & Private |
| Diagnosing Post-Cesarean Surgical Site Infections in Rural Rwanda: Development, Validation, and Field Testing of a Screening Algorithm for Use by Community Health Workers | Cherian | 2020 | Surgical Infections | Rwanda | Rural | Public |
